# End-to-End Automated Latent Fingerprint Identification With Improved DCNN-FFT Enhancement

**DOI:** 10.3389/frobt.2020.594412

**Published:** 2020-11-30

**Authors:** Uttam U. Deshpande, V. S. Malemath, Shivanand M. Patil, Sushma V. Chaugule

**Affiliations:** ^1^Department of Electronics and Communication Engineering, KLS Gogte Institute of Technology, Belagavi, India; ^2^Department of Computer Science and Engineering, KLE Dr. M. S. Sheshgiri College of Engineering, and Technology, Belagavi, India

**Keywords:** AFIS, DCNN, FFT, frequency enhanced map, FVC2004, NIST SD27

## Abstract

Automatic Latent Fingerprint Identification Systems (AFIS) are most widely used by forensic experts in law enforcement and criminal investigations. One of the critical steps used in automatic latent fingerprint matching is to automatically extract reliable minutiae from fingerprint images. Hence, minutiae extraction is considered to be a very important step in AFIS. The performance of such systems relies heavily on the quality of the input fingerprint images. Most of the state-of-the-art AFIS failed to produce good matching results due to poor ridge patterns and the presence of background noise. To ensure the robustness of fingerprint matching against low quality latent fingerprint images, it is essential to include a good fingerprint enhancement algorithm before minutiae extraction and matching. In this paper, we have proposed an end-to-end fingerprint matching system to automatically enhance, extract minutiae, and produce matching results. To achieve this, we have proposed a method to automatically enhance the poor-quality fingerprint images using the “Automated Deep Convolutional Neural Network (DCNN)” and “Fast Fourier Transform (FFT)” filters. The Deep Convolutional Neural Network (DCNN) produces a frequency enhanced map from fingerprint domain knowledge. We propose an “FFT Enhancement” algorithm to enhance and extract the ridges from the frequency enhanced map. Minutiae from the enhanced ridges are automatically extracted using a proposed “Automated Latent Minutiae Extractor (ALME)”. Based on the extracted minutiae, the fingerprints are automatically aligned, and a matching score is calculated using a proposed “Frequency Enhanced Minutiae Matcher (FEMM)” algorithm. Experiments are conducted on FVC2002, FVC2004, and NIST SD27 latent fingerprint databases. The minutiae extraction results show significant improvement in precision, recall, and F1 scores. We obtained the highest Rank-1 identification rate of 100% for FVC2002/2004 and 84.5% for NIST SD27 fingerprint databases. The matching results reveal that the proposed system outperforms state-of-the-art systems.

## Introduction

It has been more than a century that fingerprints have been used as a reliable biometric in person identification (Lee and Gaensslen, 1991; Newham, 1995). Ten-print (rolled/plain) and latent fingerprint searches are the most popularly used fingerprint matching strategies. Rolled fingerprint images are acquired by rolling fingerprints from one side to another to capture ridge information. Rolled fingerprints are capable of registering 100 minutiae and possess large skin distortions, whereas plain fingerprint images are obtained by pressing a fingertip against a flat paper surface or a scanning device. Plain fingerprints can register about 50 minutiae because of a small finger capture area and possess low skin distortions. Since 1893, latent fingerprints (Maltoni et al., 2009) have been used as one of the most crucial forensic evidence in a criminal investigations. Latent fingerprints are unintentionally left-over impressions lifted from surfaces of objects.

**Figure 1:**
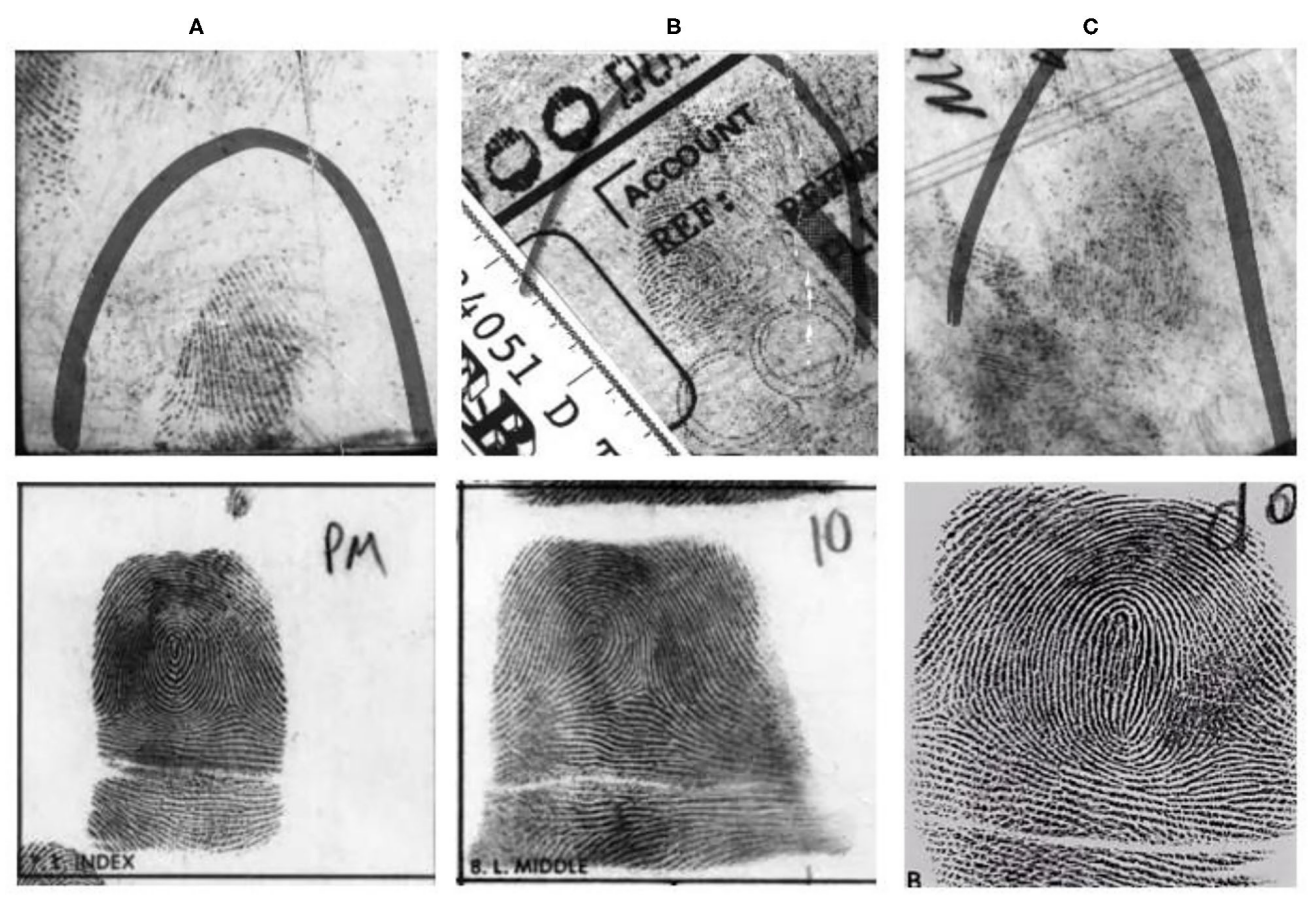
Examples of low-quality latent fingerprints from the NIST SD27 (Garris and Mccabe, 2000) database (latent fingerprints at the top and their respective true-mates at the bottom) **(A)** Original latent classified as good and its true mate **(B)** Original latent classified as bad and its true mate **(C)** Original latent classified as ugly and its true mate.

The primary difficulty with latent fingerprints identification is that it is very difficult to analyze because of its poor quality (See Figure 1). Generally, rolled/slap fingerprints are acquired under careful supervision, whereas latent prints are lifted from the surface of objects, e.g., from crime scenes, and result in poor quality ridges with the presence of complex background noise. The most challenging ability of the latent fingerprint identification system is to establish a credible link between the partial prints obtained from crime scenes with a suspects' previously enrolled fingerprints (See Figure 1) present in a large database. The challenge arises because of the poor quality latent, partial fingerprint capture area, smudged ridges, and greater non-linear skin distortions. NIST SD27 (Garris and Mccabe, 2000) is a latent fingerprint criminal database. The database is classified into good, bad, and ugly fingerprints based on the quality of fingerprints. Figures 1A–C show latent fingerprints (at the top) obtained from crime scenes and their corresponding true mates (at the bottom).

In automated fingerprint identification, a computer is used to match fingerprints against a database of known or unknown fingerprints. The Automated Fingerprint Identification Systems (AFIS) is primarily used by law enforcement agencies for a criminal investigation, to identify an unknown suspected criminal's fingerprints against the fingerprints in a large database. On the other hand, automated fingerprint verification is recognizing a known person from a relatively small fingerprint database typically in applications such as attendance and access control systems.

Before AFIS was introduced, latent fingerprints were manually matched against the actual rolled/plain fingerprints by latent examiners using an ACE-V (Ashbaugh, 1999) (Analysis, Comparison, Evaluation, and Verification). In the latent fingerprint scenario, manual matching of an unknown latent fingerprint against a large fingerprint database is a tiring process and practically not feasible—manual intervention in fingerprint matching can lead to errors.

## Literature Survey

The topic of automatic fingerprint identification is one of the most popularly searched and studied topics in the biometrics system in the past 50 years (Jain et al., 2016). The evolution of Automated Fingerprint Identification Systems (AFIS) has helped to significantly improve the speed and accuracy of rolled or plain fingerprint identification using a large fingerprint database. The Fingerprint Vendor Technology Evaluation (FpVTE) (Wilson et al., 2004) in 2003 showed that the results of commercial fingerprint matches achieved an impressive Rank-1 identification rate of more than 99.4% on a database of 10,000 plain fingerprint images. On the other hand, AFIS, developed for latent print to rolled fingerprint matching, continues to pose more challenges due to the poor ridge quality, and complex background noise of the latent print. The accuracy in such systems remains low compared to rolled/plain fingerprint matching systems.

Due to the poor latent print, most of the existing latent identification modules in AFIS work with semi-automatic configuration. In this process, a forensic expert first manually marks minutiae features in a latent, obtains the candidate list from search results, and identifies the true fingerprint from the list. Despite manual intervention in feature marking and matching stage, matching accuracy has not reached a satisfactory level. FBI's IAFIS reported a Rank-1 identification rate of 54% on a database of about 40 million latent fingerprints (Jain et al., 1997).

Most of the AFIS make use of more reliable minutiae features (Dvornychenko and Garris, 2006) for fingerprint matching. Accurate minutiae extraction results decide the match performance of the AFIS and are considered to be a very critical step in matching. Minutiae extraction methods are classified into two categories. The conventional extraction method involves extracting handcrafted features from the fingerprint domain-knowledge. Whereas the deep learning method is used to learn the features from data automatically. Minutiae extraction from rolled or slap fingerprints have produced good matching results.

Before extracting minutiae (Jain et al., 1997, 2008; Feng, 2008), the AFIS makes use of pre-processing stages such as obtaining a Region of Interest (ROI), ridge extraction, ridge enhancement, and ridge thinning. This approach works well with good quality rolled/plain fingerprints. For poor quality rolled/plain or latent fingerprints, this approach provides inaccurate minutiae location and orientation, without improving the quality of the fingerprints.

Researchers (Jain et al., 2008; Jain and Feng, 2011) concluded that the latent fingerprint identification accuracy was improved by using manually marked minutiae, ROI, and ridge flow. They further reported improvement in accuracy by using additional manually marked extended features, ridge-spacing, and skeleton. Simple steps like ridge extraction, thinning, and minutia extraction (Ratha et al., 1995) were proposed. These methods suffer due to the presence of background noise. To overcome this, Gabor filters (Gao et al., 2010; Yoon et al., 2011) are used to enhance the latent images and in turn, overcome the influence of background noises. These methods perform better than (Jain et al., 1997) but are only able to extract low-level ridge features with the help of handcrafted methods. Overall, ridge patterns in latent fingerprints suffer in the presence of background noises. Therefore, it is very difficult to extract handcrafted features with complex background noises. As discussed earlier, manual intervention in marking the features is a tiring and time-consuming process and is not feasible when a large fingerprint database is involved.

To reduce human involvement, some level of automation was introduced in the fingerprint identification process. Automatic ROI cropping (Choi et al., 2012; Zhang et al., 2013; Cao et al., 2014; Nguyen et al., 2018a), ridge-flow estimation (Feng et al., 2013; Cao et al., 2014, 2015; Yang et al., 2014), and ridge-enhancement (Feng et al., 2013; Li et al., 2018; Prabhu et al., 2018) methods were proposed by various researchers. The Descriptor-Based Hough Transform (DBHT) (Paulino et al., 2013) was proposed to align and match the fingerprints. The orientation field was reconstructed using minutiae marked by latent examiners. Another state-of-the-art matcher called the Minutia Cylinder-Codes (MCC) based indexing algorithm (Medina-Pérez et al., 2016) was proposed. MCC performs fingerprint alignment at the local level through Hough transform and to improve the matching accuracy, clustering based on Minutiae Cylinder Codes (MCC), M triplets, and Neighboring Minutiae-Based Descriptors (NMD) was proposed. The highest rank-1 accuracy of 82.9% was reported by the NMD clustering algorithm. Some researchers (Tang et al., 2016, 2017; Darlow and Rosman, 2017; Nguyen et al., 2018b) believed that the learning-based approaches using deep networks will have a better ability to extract minutiae features from latent fingerprint images. These deep learning approaches only concentrate on a particular or set of methods used in AFIS. These proposed methods do not build a complete end-to-end AFIS. Several researchers (Cao et al., 2018a,b; Cao et al., 2019) proposed solutions to build end-to-end AFIS. An automated s Convolutional Neural Networks (ConvNet) based ridge-flow, ridge-space, minutiae extraction, minutiae-descriptor extraction, extract complementary templates, and graph-based matching was proposed (Cao et al., 2018b). This method achieved the highest Rank-1 identification accuracies of 64.7% for the NIST SD27, against a background database of 100K rolled fingerprints. However, this method depends on manually marked ROI and it consumes more template match time. To overcome these problems, a texture template-based approach was proposed (Cao et al., 2018a). To make up for the lack of a sufficient number of minutiae in poor quality latent prints, virtual minutiae was introduced to improve the overall match accuracy. It resulted in the improvement of Rank-1 identification accuracy of 68.2% with a 10K gallery database. To further improve the performance proposed work (Cao et al., 2018a), an automated ROI-cropping, preprocessing, feature extraction, and feature matching was developed (Cao et al., 2019). For every minutia, a 96-dimensional descriptor is extracted from its neighborhood. To improve the computation, the descriptor length for virtual minutiae is reduced to 16 using product quantization. Highest Rank-1 identification accuracy (rank level fusion) of 70% was achieved for the NIST SD27 latent fingerprint database with 100K rolled prints. This method suffers from improper cropping with dry laments and the match accuracy is less compared to the state-of-the-art descriptor-based matcher. Deshpande et al. (2020a) proposed a deep network based end-to-end matching model called “CNNAI”. The system achieved highest Rank-1 identification rate of 80% for FVC2004 fingerprints and 84% for NIST SD27 databases. However, the system failed to identify genuine minutiae in latent fingerprints due to broken and inconstant ridges.

**Figure 2:**
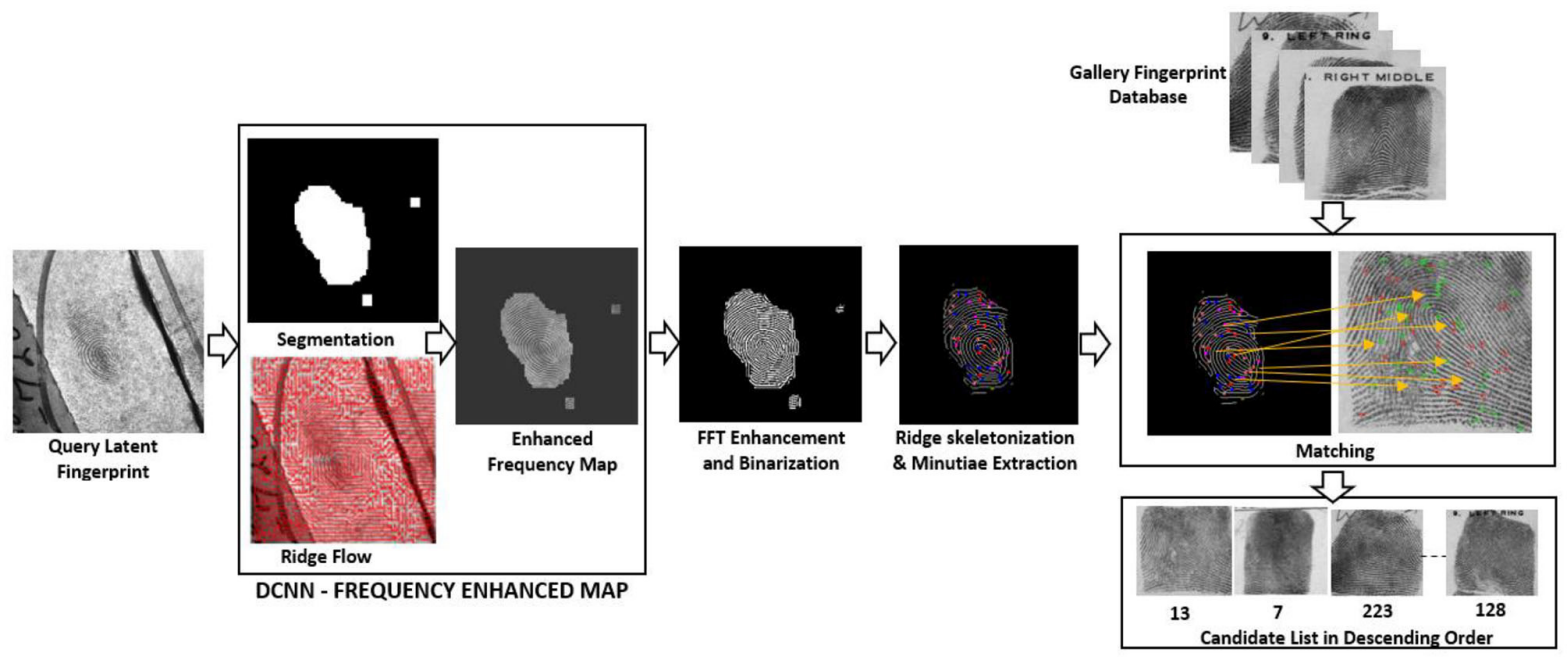
Proposed end-to-end automated latent fingerprint identification system.

**Figure 3:**
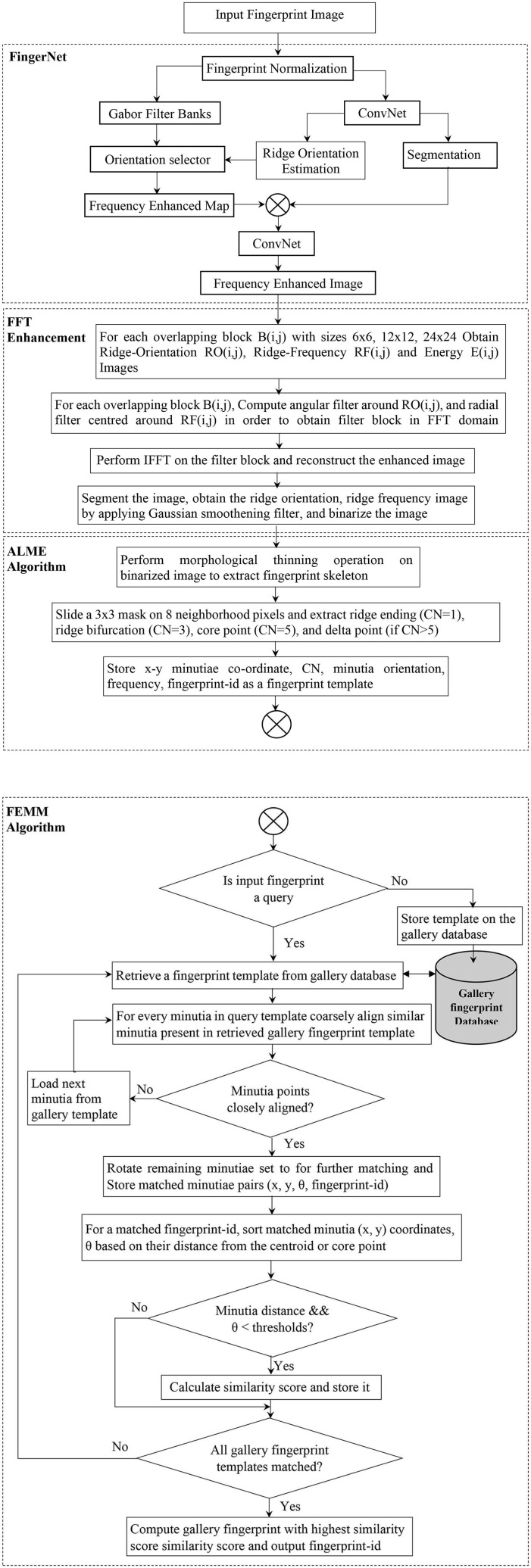
Fingerprint enhancement, minutiae extraction, and matching process involved in developing our proposed end-to-end automated latent fingerprint identification system.

In this paper, we have proposed the development of an automated end-to-end system that pre-processes, enhances, extracts the minutiae, and outputs the candidate list. In section Literature Survey we report work carried out by different researchers in this field. In section Automated Latent Fingerprint Pre-processing and Enhancement Using DCNN and FFT Filters we generate the enhanced frequency map from a Deep Convolutional Neural Network (DCNN) model. Moreover, we enhance the image using blocks of FFT enhancement filters to extract all possible ridge structures. Section For Automated Minutiae Extraction and Matching deals with the minutiae extraction, minutiae template generation, template matching, and outputting the candidate list. In section Result and Discussion we discuss the obtained result and conclude the paper. The proposed end-to-end automated latent fingerprint identification system is shown in Figure 2. We have reported our results on FVC2002 (FVC2002, 2012), FVC2004 (FVC2004, 2012) plain fingerprint, and NIST SD27 (Garris and Mccabe, 2000) latent fingerprint databases. The overall process involved in developing an end-to-end automated latent fingerprint identification system is shown in Figure 3. We compare the precision, recall, and F1-score which are minutiae extraction measures with the state-of-the-art minutiae extraction algorithms (Watson et al., 2007; Verifinger, 2010; Tang et al., 2017; Nguyen et al., 2018b). Later, we compare our Rank-1 identification accuracy with the state-of-the-art algorithms (Medina-Pérez et al., 2016; Cao et al., 2018b, 2019).

## Automated Latent Fingerprint Pre-processing and Enhancement Using DCNN and FFT Filters

**Figure 4:**
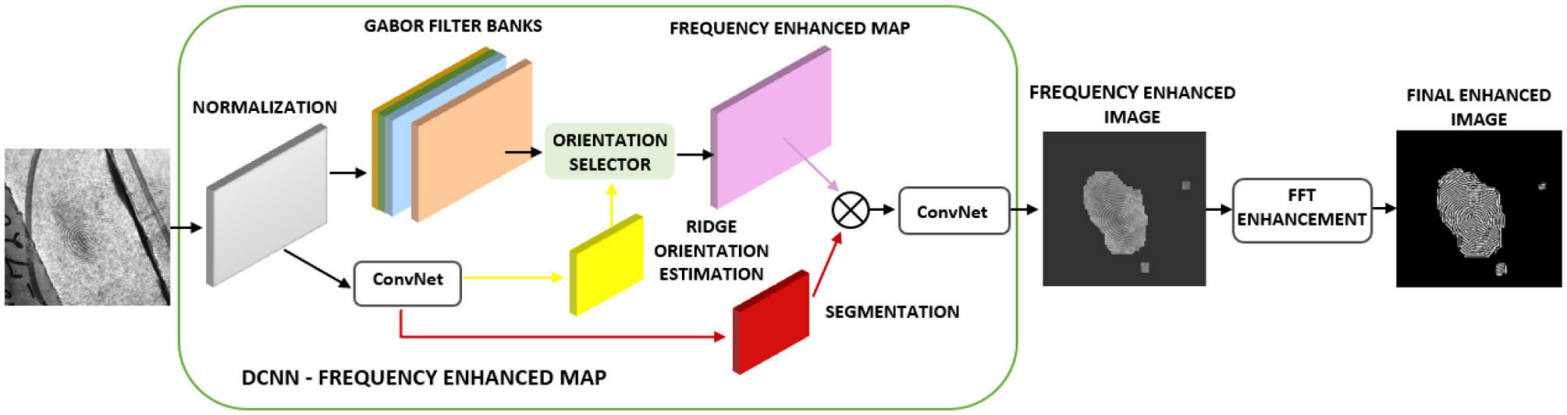
Automated latent fingerprint pre-processing and enhancement module of end-to-end automated latent fingerprint identification system.

The latent fingerprint pre-processing stage is made up of fingerprint image normalization, orientation estimation, segmentation, and enhancement steps. The performance of the minutiae extraction algorithm completely depends on the preprocessing stage and the quality of the input fingerprint images. Initially, we use part of the DCNN layers of FingerNet (Tang et al., 2017) to obtain the enhanced frequency map. Later, we enhance the ridge structure to assist good minutiae extraction. Minutiae extracted by FingerNet did not produce a good number of minutiae and this affected the performance of the extraction model as well as the matching. Thus, we use Layers of DCNN to remove the background noise and to extract the important ridge information. Further there is a need to process the images obtained from DCNN layers. To achieve good enhancement, we use FFT enhancement filters after producing an enhancement map from DCNN layers. The complete automated latent fingerprint pre-processing and enhancement block diagram is shown in Figure 4. The steps are explained next.

### Normalization

This step normalizes the overall global structure of the input image [I(i,j)]. This is achieved by reducing input images to a fixed mean (M) and variance (VAR0). This step performs a pixel-wise operation (Hong et al., 1998) to normalize the contrast and brightness of the image without changing the ridge structure. The normalized image G(i,j) is defined as,

(1) $$G\text{​}\left(i,j\right)=\{\begin{array}{c} M_{0}+\sqrt{\frac{VAR_{0}({\left(I\left(i,j\right)-M\right)}^{2}}{VAR}},\;I\left(i,j\right)>M\\ M_{0}-\sqrt{\frac{VAR_{0}({\left(I\left(i,j\right)-M\right)}^{2}}{VAR}},\;Otherwise \end{array}$$

### Orientation Field Estimation

The fingerprint image contains ridge distribution in different parts of the fingerprint. To determine the dominant direction of the ridges, this step is implemented. This is a very important step and any errors introduced here will get propagated into the next stages. Gradient and sum of windowed computations are replaced with convolutional operations (Ratha et al., 1995) and it is transformed as,

(2) $$\begin{array}{r} \nabla_{i}I=I\;*\;M_{i},\;\nabla_{j}I=I*\;M_{j},\;G_{ij}=\left(\nabla_{x}I\cdot \nabla_{y}I\right)*\;O_{w},\\ G_{ii}={\left(\nabla_{i}I\right)}^{2}*\;O_{w},\\ G_{jj}={\left(\nabla_{j}I\right)}^{2}*\;O_{w},\\ \theta =90+\frac{1}{2}atan2\left(2\cdot G_{ij},G_{ii}-G_{jj}\right) \end{array}$$

Here, ∇i and ∇j are the “i” and “j” gradients computed using “Sobel Masks” (Mi and Mj) for the input image I. “*” is a convolutional operator, and “Ow” is a matrix of one's with the size of wxw. atan2(j, i) is used to calculate the arc-tangent of the two variables j and i for their quadrant. “θ” is orientation field output. We use “ConvNet” with three convolutional pooling blocks. Each convolutional pooling block contains a pooling layer after a few convolutional blocks. Each convolutional block is made up of a convolution layer followed by a BatchNorm (Ioffe and Szegedy, 2015) and a PReLU (He et al., 2015) layer. Further, we use a multi-scale resolution (ASPP layer) and a parallel orientation regression on each feature map (Tang et al., 2017) to calculate maximum ridge orientation (θmax).

### Segmentation

The coherence (Bazen et al., 2001) gives a good response to the gradients that are pointing in the same direction. Fingerprints contain parallel ridge structures, and the coherence produces a good response to the foreground ridge information compared to the background noise. In a window “w” over pixels, the coherence, mean, and variance are computed as:

(3) $$\begin{array}{r} oh=\frac{\sqrt{{\left(G_{ii}-G_{jj}\right)}^{2\;}+4\cdot {G_{ij}}^{2}}}{G_{ii}+G_{jj}},\\ Mean=\frac{I*\;O_{w}}{w^{2}},\\ Var=\frac{{\left(I-Mean\right)}^{2}*\;O_{w}}{w^{2}},\\ Seg=w\;*\;\left[\text{Coh},\text{Mean},\text{Var}\right]+\beta , \end{array}$$

Where “w” is the length of the local window, and “β” is the classifier's parameter. To implement a segmentation map using deep layers, the entire multi-scale feature maps are shared with an orientation estimation part as discussed in Orientation Field Estimation. To predict the pixel within the region of interest (ROI), a segmentation score with the size of “H/8 × W/8” is chosen.

### Enhancement

Because of frequency selective characteristics, Gabor enhancement (Bernard et al., 2002) is widely used in fingerprint identification applications. To obtain ridge frequency “ω” and ridge orientation “θ,” convolution operations are conducted on a fingerprint block. Gabor enhanced fingerprint block GE(i,j) for a pixel (i,j) in an image I am calculated as,

(4) $$\begin{array}{r} GE\left(i,\;j\right)\;=\;\left(I\;*\;g\omega ,\theta \right)\left(i,\;j\right) \end{array}$$

(5) $$\begin{array}{r} GE\left(i,\;j\right)\;=\;A\left(i,j\right)\cdot e^{i\emptyset \left(i,j\right)} \end{array}$$

where A(i, j) and iϕ(i0, j0) is the amplitude and phase components of the Gabor enhanced block. ϕ(i0, j0) is taken as the final enhanced results. The orientation map discussed in section Orientation Field Estimation from the orientation mask is directly multiplied by Gabor filter banks.

To obtain a Gabor filter bank, Gabor filters with “N” discretized parameters are generated, respectively, and the set of filtered images are obtained by convolving with Gabor filters. The orientation selector is a mask that will choose the appropriate enhanced blocks from the filter banks. The final frequency enhanced map is obtained by convolving the Gabor filter bank images with enhanced blocks. This frequency enhanced map is further enhanced using FFT filters.

**Figure 5:**
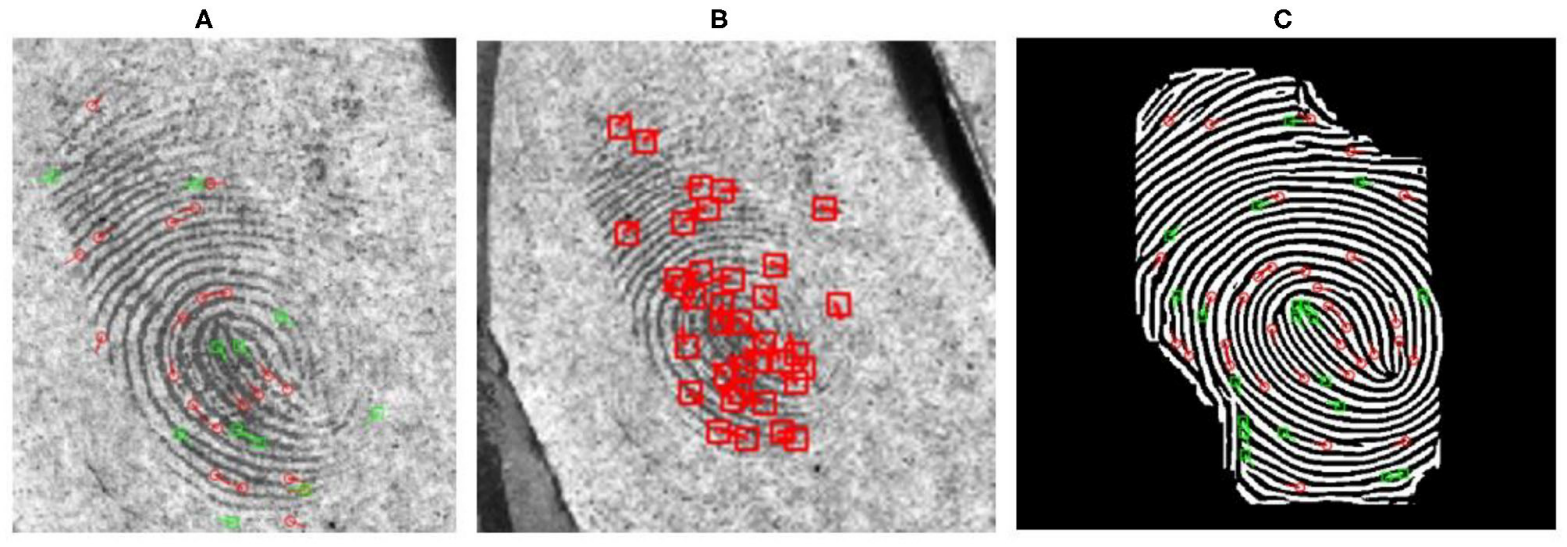
Minutiae extraction before and after FFT enhancement **(A)** Minutiae extracted from FpMV minutiae detection application^1^ **(B)** Minutiae extracted from FingerNet (Tang et al., 2017) **(C)** Minutiae extracted after FFT enhancement using FpMV minutiae detection application.

Figure 5A shows the minutiae detected using NIST FpMV minutiae detection application^1^. When the minutiae features are extracted (without the FFT enhancement) using FingerNet (Tang et al., 2017) (See Figure 5B), the system failed to extract many genuine minutiae while producing few spurious minutiae compared to the fingerprint shown in Figure 5A. Any fingerprint recognition system can deal with the presence of few spurious minutiae, but matching performance is affected when the system fails to extract a sufficient number of reliable minutiae from the latent fingerprints which already contain partial ridge information. To overcome this problem we therefore further enhance the image using FFT filter banks. Figure 5C shows the minutiae extracted from the FFT enhanced image and it can be observed that the FpMV detection application detects a greater number of ridge structures and genuine minutiae compared to Figures 5A,B.

### FFT Enhancement

**Figure 6:**
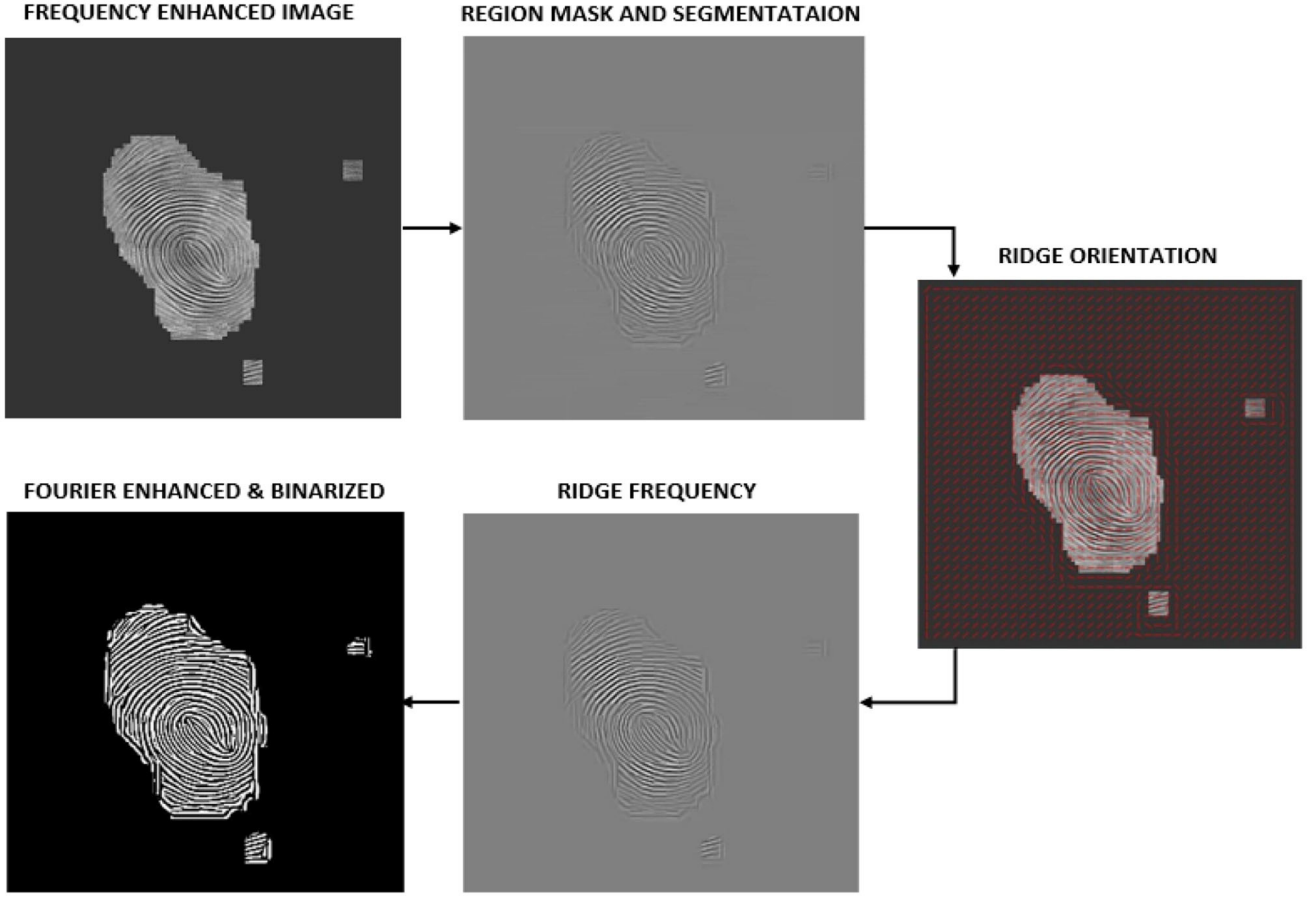
Steps used in implementing automated FFT enhancement algorithm.

Here we enhance the frequency enhanced map obtained from DCNN layers using Fourier filters (Chikkerur et al., 2007). In this process, the image is divided into small overlapping windows. To obtain the ridge frequency and orientation, these small window regions are analyzed. The energy distribution is used as a region-mask to separate foreground fingerprint information with the background noise. The Fourier spectrum of this small region is analyzed, and probabilistic estimates of the ridge frequency and ridge orientation are obtained. Based on the analysis the energy map is used as a region mask to distinguish between the fingerprint and the background regions. Based on the ridge orientation information, an angular coherence image (Rao, 1990) is obtained. This results in contextual information and helps in filtering each window in the Fourier space. Butterworth Band pass and root filters are used to enhance the ridges on the overlapping blocks. Finally, the enhanced image is obtained by inverting each window. Figure 6 shows the steps used in implementing the automated FFT enhancement. These steps are explained next.

#### Region-Mask and Segmentation

Latent fingerprints contain a complex and noisy background with little ridge information. This results in little frequency in the Fourier space. Let, E(i,j) define the energy content of the respective block.

(6) $$\begin{array}{r} E\left(i,\;j\right)\;=\text{log}\left\{\int_{r}\int_{\theta }|F(r,\theta {|}^{2}\right\} \end{array}$$

The fingerprint is segmented from the foreground and background by automatically thresholding (Otsu, 1979) the energy image. The image is further processed to obtain the connected components.

#### Ridge-Orientation

Let the orientation “θ” be a random variable with probability density function P(θ). The orientation values are obtained by performing a vector averaging. To resolve orientation difficulty between the orientation of ±180°, sin(2θ) and cos(2θ) are used.

(7) $$\begin{array}{r} E\left\{\theta \right\}\;=\frac{1}{2}{\text{tan}}^{-1}\left\{\frac{\int_{\theta }P\left(\theta \right)\sin\left(2\theta \right)d\theta }{\int_{\theta }P\left(\theta \right)\cos\left(2\theta \right)d\theta }\right\} \end{array}$$

Since the latent fingerprints contain poor ridge structures, we estimate the ridge orientation from its immediate neighborhood. The final resulting orientation image O(i,j) is further smoothened using the Gaussian smoothening-kernel (W). Smoothening kernel W(i,j) of size 3 × 3 is used over a 5 × 5 or 7 × 7 kernel.

(8) $${\text{O}}^{\prime }(\text{i},\text{ j})\;=\frac{1}{2}\{{\text{tan}}^{-1}\frac{\sin\;(2O{\left(i,j\right)}^{*}W(i,j))}{\cos\;(2O{\left(i,j\right)}^{*}W(i,j))}\}$$

#### Ridge-Frequency

Similar to ridge orientation, the ridge frequency is calculated as,

(9) $$\begin{array}{r} \text{E}\left\{\text{r}\right\}\;=\int_{r}P\left(r\right)rdr \end{array}$$

Because of simple plain smoothening, ridge-frequency obtained at the boundaries of the fingerprint can propagate errors over the complete image. To overcome this, the modified smoothened filter is obtained by,

(10) $$\begin{array}{r} F^{\prime }\left(i,\;j\right)\;=\frac{\sum_{u=i-1}^{i+1}\sum_{v=j-1}^{j+1}F\left(u,v\right)W\left(u,v\right)I\left(u,v\right)}{\sum_{v=j-1}^{j+1}W\left(u,v\right)I\left(u,v\right)} \end{array}$$

“W” represents the Gaussian smoothening-kernel with 3 × 3 size. Inter-ridge distance lies a range of 3 to 25 pixels per ridge and the ridges outside this range are considered invalid.

#### FFT Enhancement Algorithm

We use FFT filters to obtain the final enhanced image from the ridge frequency image. FFT filters are obtained by performing different operations, by analyzing the result obtained in previous steps. Enhancement at different stages of the FFT enhancement algorithm is shown in Figure 6. Complete FFT enhancement step is explained in the Algorithm 1. It can be observed that the minutiae extracted after FFT enhancement using FpMV minutiae detection software are close to the ground truth minutiae (See Figure 5B).

**Algorithm 1:** FFT Enhancement Algorithm.

Input ← Frequency enhanced image I(i,j)
Output ← FB(i,j) FFT enhanced and binarized image
for each image I(i,j)
for each overlapping block B(i,j) with sizes 6x6, 12x12, 24x24
Remove DC components in B, multiply with spectral window ‘w', filter (F) ridges separated between 3 to 25 pixels using (32x32) Band pass Butterworth filter (F = FFT(B)), and perform root filtering (R = 0.5) on F.
Obtain values of Ridge-Orientation RO(i,j), Ridge-Frequency RF(i,j) and Energy Image E(i,j)
Construct enhanced image maps using,
RO‘(i,j) ← Smoothening Ridge Orientation map from RO(i,j).
RF‘(i,j) ← Diffusing Ridge Frequency map from RF(i,j).
C(i,j) ← Computing coherence image using RO‘(i,j).
RM(i,j) ← Computing Region Mask by thresholding E(i,j).
F_A_ ← Computing angular frequency around RO(i,j).
F_R_ ← Computing radial filter centered around RF(i,j).
FFT ← F* F${}_{\text{A}}^{*}$F_R_
I‘(i,j) ← IFFT(F) Reconstruct the enhanced image from F
end for
Obtain,
RS(i,j) ← Segmented image by applying Region Mask RM(i,j) over I‘(i,j).
ROG(i,j) ← Ridge-Oriented image by applying Gaussian smoothening-kernel over RS(i,j).
RFE(i,j) ← Ridge Frequency Enhanced image by masking non-ridge regions and orientation
filtering over ROG(i,j).
FB(i,j) ← Binarize the Frequency enhanced image RFE(i,j).
end for

After the final enhancement step, we obtain the enhanced and binarized image ready for feature extraction. NIST's FpMV detection software is a tool used to observe minutiae and cannot be directly integrated into the proposed end-to-end. We therefore propose an automated minutiae extractor and matching to achieve this operation.

## For Automated Minutiae Extraction and Matching

Minutiae-based matching methods are the most commonly preferred method (Ratha and Bolle, 2003) because of their uniqueness (no two fingerprints can have similar minutiae patterns and minutiae do not change throughout its lifetime) and ease of extraction. We discuss minutia-based extraction and matching in this section. Minutiae present in the gallery fingerprints are extracted and stored as a set of minutiae belonging to a particular fingerprint with the fingerprint ids. For a query fingerprint our proposed minutiae extractor performs fingerprint alignment to retrieve minutiae pairings between both the fingerprints. Our proposed matcher computes the similarity between the query and gallery fingerprints, searches the top 20 similar fingerprints, and finally outputs the candidate list based on the match score. These steps are explained in detail next.

### Automated Latent Minutiae Extractor (ALME)

After the frequency enhanced, and the binarized image is obtained, the next step is to automatically extract the minutiae features from a skeletonized fingerprint. We propose the Automated Latent Minutiae Extractor (ALME) to achieve this task. The ALME operation is explained in Algorithm 2. To obtain an image skeleton, a morphological thinning operation is performed on the ridge structure to reduce their size by 1-pixel thickness. This is done by deleting pixels present at the edge of ridgelines until the pixel size is reduced to 1 pixel. Skeletonization of low-quality fingerprint results in ridge breaks, bridges, irregular ridge endings, and can introduce false minutiae. Our proposed enhancement method discussed in section Automated Latent Fingerprint Pre-processing and Enhancement Using DCNN and FFT Filters overcomes these problems by improving the quality of latent fingerprints (See Figure 5).

**Algorithm 2:** Automated Latent Minutiae Extractor (ALME) Algorithm.

Input ← Frequency enhanced and binarized image FB(i,j)
Output ← Extracted fingerprint templates (x, y, CN, $\hat{\text{C}}$θ, f, fingerprint-id)
for each image FB(i,j) do
Fingerprint Skeleton Skel (i,j) ← Morphological thinning operation on FB(i,j)
for each pixel in FB(i,j) do
Crossing Number (CN) ← Slide 3x3 mask to find pixels in 8 neighborhoods
if (CN == 1) then
minutia ← ridge ending
else if (CN == 3) then
minutia ← ridge bifurcation
else if (CN == 5) then
minutia ← core point
else
minutia ← delta point
end if
Store fingerprint template ← x-y minutiae co-ordinate, CN, minutia orientation, frequency, fingerprint-id
end for
end for

After thinning, the binary image containing pixel “p” is analyzed to locate the minutiae location. Crossing-number (Rutovitz, 1966) is obtained by considering the 8-neighborhood pixels (in a 3 × 3 window with p as center) circularly traversed in an anti-clockwise manner.

(11) $$\begin{array}{r} cn\left(p\right)=\frac{1}{2}\sum_{i=1\ldots 8}|val(p_{\left(\text{imod}8\right)}-\text{val}\left(p_{i-1}\right)| \end{array}$$

Here, cn(p) represents the crossing number of a pixel “p” and val(p)∈{0,1} is the binary value. The crossing number identifies the minutiae pixel location as ridge ending (cn=1), bifurcation (cn=3), core point (cn=5), and delta point (cn>5) in the thinned image.

**Figure 7:**
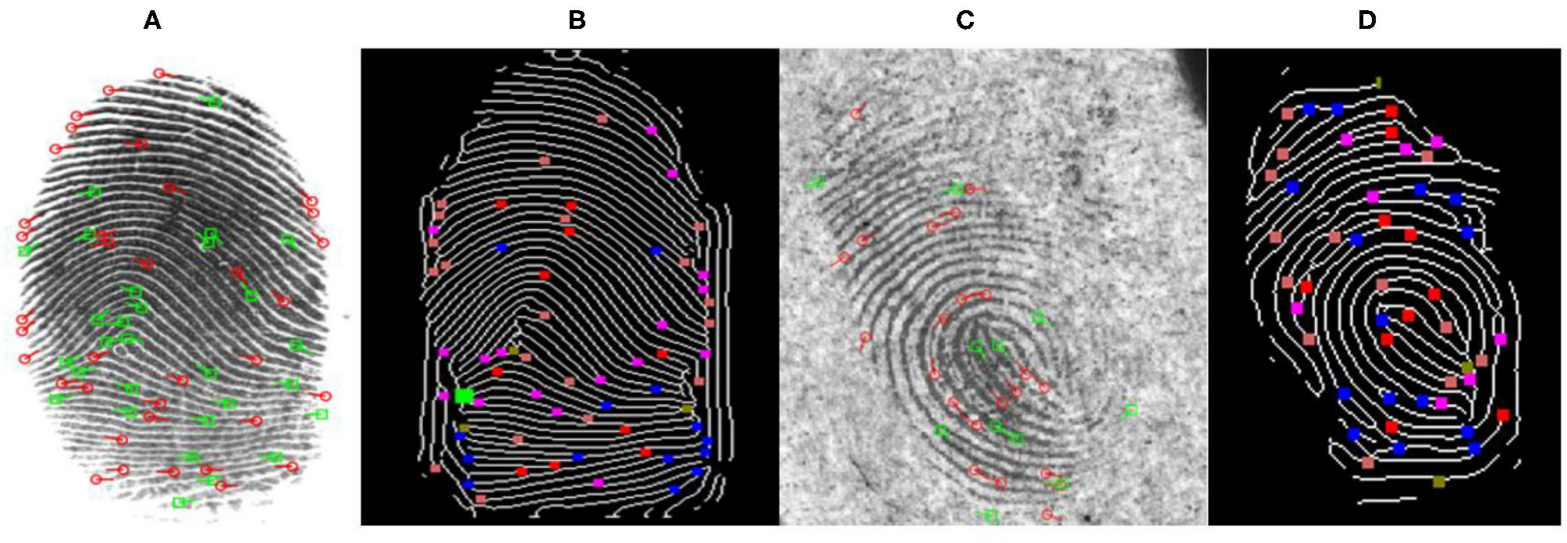
Minutiae extracted from FVC2004 (FVC2004, 2012) and NIST SD27 databases (Garris and Mccabe, 2000) **(A)** Original plain FVC2004_DB1 extraction using FpMV **(B)** Proposed skeletonization and minutiae extraction of fingerprint seen in **(A)** after Fourier enhancement **(C)** Original NIST SD27 extracted from FpMV **(D)** Proposed skeletonization and minutiae extraction of latent fingerprint seen in **(C)** after Fourier enhancement.

It can be seen that many false (spurious) minutiae appear in the original FVC2004_DB1 and NIST SD27 fingerprints when they are extracted using FpMV without enhancement (See Figures 7A,C). False minutiae are detected mainly because of broken ridges within and at the borders of fingerprints. Additionally, when these images are not enhanced, they do not reveal important ridge information and as a result they miss out important minutiae in critical regions of the fingerprint (See Figures 7A,C). After the fingerprints are enhanced using the FFT enhanced method (as discussed in section Automated Latent Fingerprint Pre-processing and Enhancement Using DCNN and FFT Filters), the broken ridges become connected and hence it removes false minutiae from fingerprints when extracted. It can be seen from Figures 7C,D that a missing ridge structure within and at the boundary of the fingerprint are visible enhancement and minutiae are detected. In Figures 7B,D core-point is indicated by green, delta or lower core-points with gold, bifurcations with blue [for θ ∈ (0°-180°)] and purple [for θ ∈ (180°-360°)], and ridge-endings with orange [for θ ∈ [0°-180°)] and red [for θ ∈ (180°-360°)]. Overall, compared to the original fingerprints the minutiae extracted after frequency enhancement produced tolerable false minutiae. Extracted minutiae are finally stored as templates in the form of (x, y, CN, θ, f). x, y represents minutiae coordinates, Crossing Number (CN) indicates the type of minutia (ridge ending, bifurcation, etc.). It is indicated in different colors as discussed above. “θ” indicates the value of ridge orientation concerning reference minutia. “f” indicates ridge frequency. This value will be 0 or 1 because of binarization. The extracted minutiae using ALME for gallery fingerprints are stored as minutiae templates with fingerprint id's in the database. For a given input query fingerprint the minutiae are extracted by ALME and the matching is performed. Fingerprint matching is explained next.

### Frequency Enhanced Minutiae Matcher (FEMM)

In this process, minutiae from gallery fingerprints are extracted and are stored as templates in the database. A template for the input query fingerprint is obtained and two fingerprint templates are compared based on the degree of similarity between them. The similarity score between 0 and 1 indicates the percentage of closeness to its original reference fingerprint. The matching decision depends on the selected threshold value. The performance of any matcher is measured in terms of matching accuracy and response time. These performance metrics change according to the fingerprint application.

Fingerprint matchers are classified into three categories:

- Correlation-based: To perform matching, fingerprint images are superimposed and the correlation between the corresponding pixels at different displacement and rotations is calculated. This method greatly relies on accurate fingerprint alignment before matching.
- Minutia -based: The minutiae from two fingerprints are extracted and stored to find the maximum number of matching pairs in the sets. Accurate minutiae detection is key in this method.
- Non-minutiae based: The matcher uses global features such as ridge-orientation and frequency to align and match two fingerprints. Global features are not affine to rotation, scale, and hence become difficult to align.

We use a minutiae-based matching on frequency enhanced images. We propose a Frequency Enhanced Minutiae Matcher (FEMM) to align and match the fingerprints. To begin, we calculate the average distance between the stored ground truth (in the gallery database) and the query fingerprint minutiae pairs to obtain the alignment error. If the average Euclidean distance between the latent minutiae pairs is less than a predefined number of pixels, then we consider it to be a correct fingerprint alignment. This alignment is mainly done to initiate the matching process as well as to remove the false minutiae. Depending on the number of corresponding match pairs between the two fingerprints, the match score is calculated. The matching process using the FEMM operation is explained in Algorithm 3.

**Algorithm 3:** Frequency Enhanced Minutiae Matcher (FEMM) Algorithm.

Input ← Query fingerprint template (x, y, CN, θ, f, fingerprint-id)
Output ← Similarity score and matching fingerprint-id
for each fingerprint template in Gallery database do
for each minutia in Query fingerprint template do
Align (δx, δy, α) ← coarsely align similar minutiae present in gallery fingerprint
template by translating (δ) /rotating (α between -5 to +5 degrees) fingerprints
if fingerprints are closely aligned then
Rotate any minutiae set to for further matching
if ((minutiae pair position && orientation) == true)
then
Store matched minutiae pairs (x, y, θ, fingerprint-id)
end if
else
Load next minutia from the fingerprint selected from gallery fingerprint
end if
end for
For a matched fingerprint-id, sort matched minutia (x, y) coordinate positions, orientation (direction)
based on their distance from the centroid or core point.
if (distance && orientation thresholds < pre-defined thresholds) then
Output similarity score
end if
end for
Matching fingerprint-id ← Gallery fingerprint with highest similarity score

#### Fingerprint Alignment

The major limitation of fingerprint alignment, especially in latent fingerprints, is that the singular or core points may not always be present, unlike good quality fingerprints. Aligning based on manually marked features is prone to developing errors and is a tiresome process. FEMM performs the fingerprint alignment based on the available minutiae pairs. Initially, minutiae structures of two fingerprint images are coarsely aligned. This is done by translating and rotating two fingerprints so that one minutia from one fingerprint closely overlaps with another similar minutia from the other fingerprint. After two fingerprints are closely aligned in specific translation and orientation, one of the minutiae sets is rotated to optimize the alignment between two minutiae sets for further minutiae matching. Minutiae pairs with close position and orientation are considered to be corresponding matched pairs.

As discussed, to overcome the problems associated with alignment, we use a method similar to the registration technique (Ratha et al., 1996). We use a Hough based two-step minutiae registration and pairing method. Here minutiae registration involves a process of aligning a pair of minutiae within a specified range using parameters < δx, δy, α >. (δx, δy) are the translation parameters and “α” is the rotation parameter. With the help of these parameters, minutiae sets are rotated and translated within parameter specified limits. Each transformation produces a score for a pair of minutiae, and we find the highest alignment transformation score to register it. Using this transformation score we align the query minutiae set with that of a fingerprint minutiae set in the gallery database. We set the value of “α” between −5 degrees to 5 degrees in 1 or 2 steps. Some rotational steps are represented as < α1, α2, α3.αk > where “k” is the number of rotations applied.

To check similar fingerprints, for every query minutiae “i” we see if,

(12) $$\begin{array}{r} \alpha_{k}+\theta_{i}=\theta_{j} \end{array}$$

Where “θi” and “θj” are the orientation parameters of ith minutia from query minutiae set and jth minutia from gallery fingerprint minutiae set. If the condition in Equation (11) is satisfied, then P (i, j, k) is set as “1” or else “0.” For values set as “1,” translation parameters (δx, δy) are calculated using the following equation.

(13) $$\begin{array}{r} \left(\delta_{x\;},\delta_{y\;}\right)=\;q_{j}-\left(\begin{array}{cc} \cos\theta & \sin\theta \\ -\sin\theta & \cos\theta \end{array}\right)*p_{i} \end{array}$$

where “qj” and “pi” are the coordinates of jth minutiae of gallery fingerprint minutiae set and ith minutiae of query minutiae set, respectively. Using the parameters < δx, δy, αk >, the query minutiae set is aligned. These aligned minutiae sets are used to compute the minutiae pairing score. Two minutiae are set to be paired only if they lie within the same bounding box and possess the same orientation. Minutiae pairing score is the ratio of the number of paired minutiae to the total number of minutiae. The i, j, k values with the highest minutiae pairing score will be used to align the minutiae set. Coordinates of aligned minutiae are obtained using the equation:

(14) $$\begin{array}{r} \left(q_{j}\right)=\;\left(\begin{array}{cc} \cos\theta & \sin\theta \\ -\sin\theta & \cos\theta \end{array}\right)\;*\;p_{i}+\left(\delta_{x\;},\delta_{y\;}\right) \end{array}$$

Aligned minutiae are stored for further investigation.

#### Fingerprint Matching

After the alignment is done, similar minutiae are stored in an order based on their distance from their centroid or core point. Minutiae orientation (direction) and coordinate (x, y) positions are used to match minutiae extracted from two fingerprints. Minutiae selected from query and gallery fingerprints are said to be matched if their distance and orientation differences are less than pre-defined threshold values. The minutiae difference and their threshold are given by,

(15) $$\begin{array}{r} T_{Dist\;}\geq \sqrt{\left(\Delta x^{2}+\Delta y^{2}\right)} \end{array}$$

where “Δx” is the difference between the x-coordinate of minutiae of two fingerprints. Similarly, “Δy” is the difference of minutiae for y-coordinate. The orientation difference and their threshold are given by,

(16) $$\begin{array}{r} T_{ori\;}\geq min\left(\left|\Delta \theta |,\;360^{{}^{\circ}}-|\Delta \theta \right|\right) \end{array}$$

where “Δθ” is the orientation difference of two minutiae under consideration.

Based on the number of matched minutiae from two fingerprints, the similarity score is computed. As discussed in Fingerprint Matching the minutiae extracted from two fingerprints are aligned and matched via shifting and rotating fingerprints. The similarity score is calculated as the maximum number of minutiae matching pairs. It is given by the formula,

(17) $$\begin{array}{r} Similarity\;score=\;\frac{{\left(\#\;total\;matched\;minutiae\right)}^{2}}{{\left(\#\;of\;matching\;minutiae\;pairs\;from\;query\;FP\right)}^{*}\;\left(\#\;of\;matching\;minutiae\;pairs\;from\;gallery\;FP\right)} \end{array}$$

## Result and Discussion

We conducted experiments on plain FVC2002 (FVC2002, 2012), FVC2004 (FVC2004, 2012), and NIST SD27 (Garris and Mccabe, 2000) latent fingerprint databases. FVC2002 and FVC2004 DB1 databases contain 80 fingerprints from 10 persons (class) with each person registering eight fingerprints. It therefore contains a total of 10 fingerprint classes in 80 fingerprints. Although FVC2004 is a plain fingerprint, it is made up of low-quality fingerprints and it will help to test our proposed methods. NIST SD27 is a criminal fingerprint database and contains 258 fingerprint images obtained from 258 persons. It forms a total class of 258 fingerprint images with 88 Good, 85 Bad, and 85 Ugly images. We conducted experiments on FVC2002, FVC2004, and NIST SD27 databases to check the performance of our proposed enhancement and minutiae extraction algorithm. To test the performance of minutiae matching algorithm, we used FVC2004 and NIST SD27 databases. We compared our results with different state-of-the-art methods. We implemented our end-to-end system using python and MATLAB on an intel i7 2.7GHz dual-core machine.

### Extraction Performance

We enhanced the low-quality fingerprint images with DCNN and FFT enhancement algorithms. To measure the accuracy of the minutiae extraction algorithm, we compared the extracted minutiae with that of the ground truth. We used precision, recall, and F-1 metrics to measure the performance. Precision is defined as the number of True Positives (TP) divided by the number of TP plus the number of False Positives (FP). In TP, the model correctly labels positive class and in the case of FP the model incorrectly labels as a positive and negative class. The model expresses the Precision as the proportion of the relevant data points.

(18) $$\begin{array}{r} \text{Precision }=\;\frac{TP}{TP+FP} \end{array}$$

“Recall” refers to the total percentage of relevant results that are correctly classified by an algorithm. “Recall” expresses the ability to find all relevant instances in a dataset. There is another metric called the “F-1” score, it is a Harmonic-Mean (H.M) of precision and recall values. We conducted experiments on a FVC2004DB1 and NIST SD27 latent database and compared our minutiae extraction results with the state-of-the-art algorithms (Watson et al., 2007; Verifinger, 2010; Darlow and Rosman, 2017; Nguyen et al., 2018b). The experiment was conducted with maximum minutiae distance, orientation of 8, and 10 pixels, respectively. Table 1 shows the comparisons of precision, and recall obtained by different methods. It can be observed that our proposed ALME performs well-compared to the state-of-the-art techniques for both FVC 2004 and NIST SD27 databases.

**Table 1:** Comparison of minutiae extraction performance (Precision, Recall, and F1-Score) by different state-of-the-art methods using FVC 2004 and NIST SD 27 Databases with setting-1 (Dist=8, Ori=10).

**Dataset**	**Methods**	**Precision**	**Recall**	**F1-Score**
FVC 2004	MINDTCT (Watson et al., 2007)	30.8%	64.3%	0.416
	VeriFinger (Verifinger, 2010)	39.8%	69.2%	0.505
	FingerNet (Tang et al., 2017)	68.7%	62.1%	0.643
	MinutiaeNet (Nguyen et al., 2018b)	79%	80.1%	0.795
	Proposed ALME	87.88%	63%	0.74
NIST SD27	MINDTCT (Watson et al., 2007)	8.3%	14.7%	0.106
	VeriFinger (Verifinger, 2010)	3.6%	40.1%	0.066
	FingerNet (Tang et al., 2017)	53.2%	49.5%	0.513
	MinutiaeNet (Nguyen et al., 2018b)	69.2%	67.7%	0.684
	Proposed ALME	76.19%	61.54%	0.681

**Figure 8:**
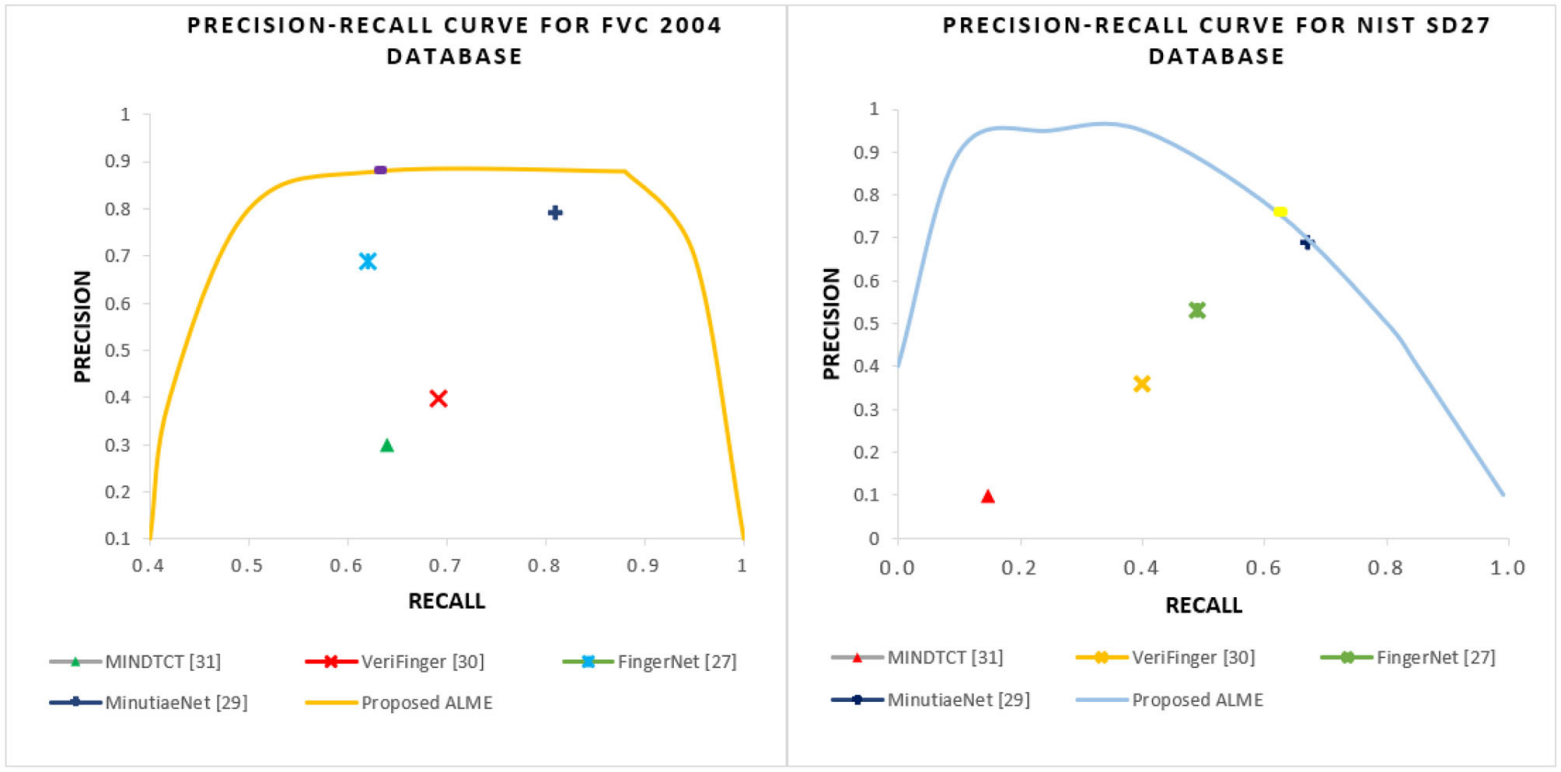
Precision-Recall curves of FVC 2004 **(Left)** and NIST SD27 **(Right)** datasets with the proposed ALME vs. state-of-the-art extraction methods.

The obtained results were found to be similar to the ground truth minutiae. The proposed frequency enhancement method algorithm can even work well with partial fingerprints and fingerprints with noisy backgrounds. Figure 8 shows a graph of the precision-recall curve for FVC2004 and NIST SD27 datasets with proposed against the reported state-of-the-art algorithms.

### Matcher Performance

**Figure 9:**
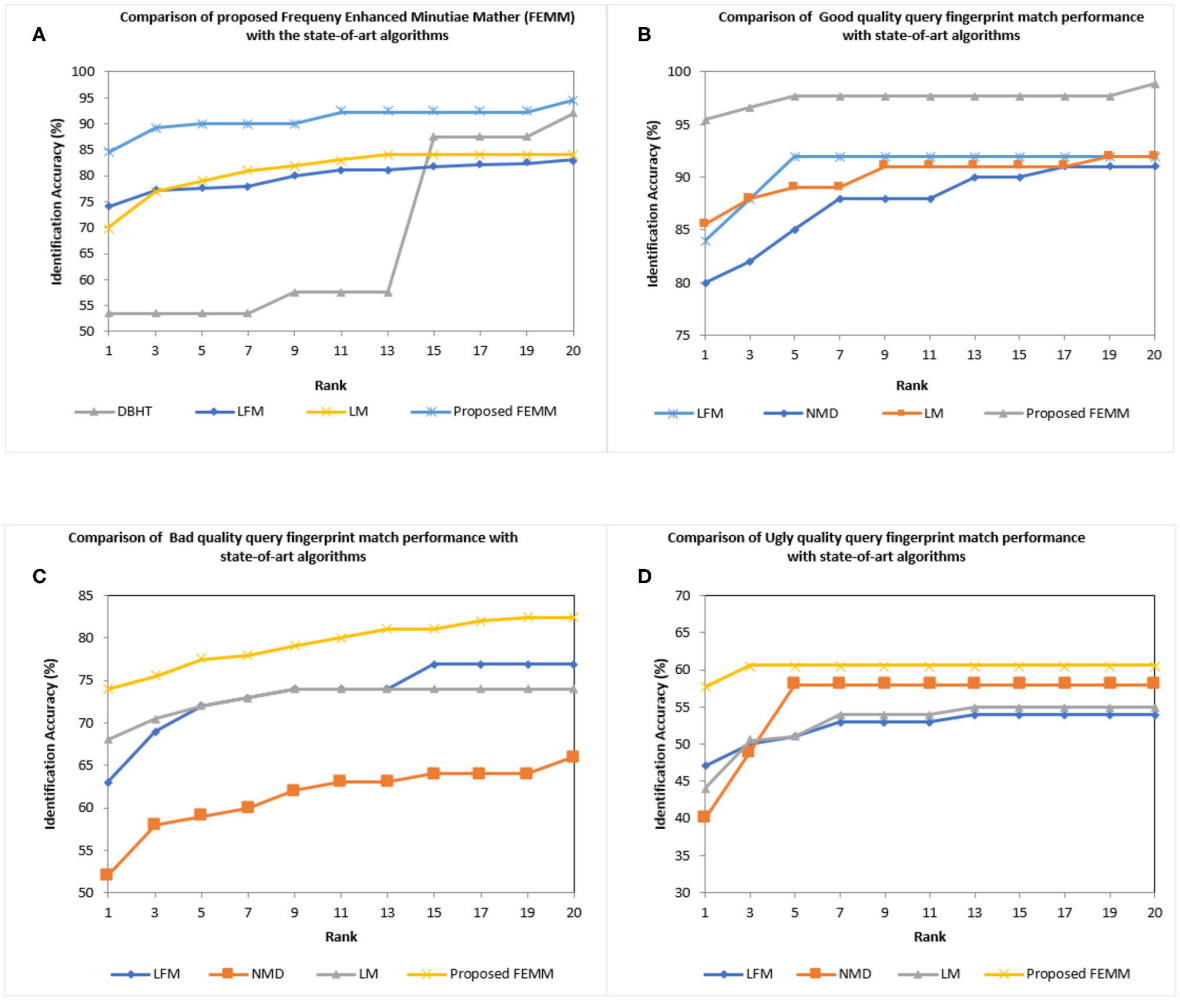
Cumulative Match Characteristic (CMC) curves comparing the performance of proposed Frequency Enhanced Minutiae Matcher (FEMM) with the reported state-of-the-art algorithms for NIST SD27 latent database **(A)** All latent **(B)** Good quality latent **(C)** Bad quality latent **(D)** Ugly quality latent.

We used Cumulative Match Characteristic (CMC) as a performance evaluation. The experiment was conducted on FVC2002, FVC2004, and NIST SD27 latent query fingerprints with close-set identification configuration (Cao et al., 2019) and we reported the top Rank-20 matching accuracy results. For FVC2002DB1 and FVC2004DB1 query fingerprints, the experiment was conducted with a gallery database of 2,560 images. These gallery fingerprints are formed from FVC2002, FVC2004 (DB2,3,4), and NIST SD4 rolled fingerprints. We obtained 100% Rank-1 identification accuracy for FVC2002 and FVC2004 query fingerprints. FVC2002/2004 are slap fingerprint databases and the quality of fingerprints is good compared to NIST SD27 latent fingerprint databases. Our proposed FEMM performed well for quality fingerprints. Next, we conducted experiments for the query NIST SD27 latent fingerprint database including all latent and different quality latent prints (good, bad, ugly). The ground truth fingerprint database of 2,258 fingerprints is made up of NIST SD27 and ND4 rolled fingerprints. Figure 9 shows the CMC curve of the proposed and the reported four state-of-the-art matchers. It can be seen that our proposed FEMM produced the highest Rank-1 identification accuracy of 84.5% compared to 70% of Latent matcher (LM) (Cao et al., 2019), 83% of NMD (Medina-Pérez et al., 2016), 74% of Latent Fingerprint Matcher (LFM) (Cao et al., 2018b) and 53.5% of DBHT. Our proposed algorithm produced Rank-20 accuracy of 94.57%. It can be seen from Figure 9A that the proposed matcher outperforms the reported state-of-the-art algorithms. We further continue our experiment with good, bad, and ugly quality latent prints. Figures 9B–D show a comparison of CMC curves for good, bad, and ugly latent prints with the state-of-the-art matchers. Our proposed matcher performed well in terms of match accuracy compared to the reported state-of-the-art algorithms.

#### FMR, FNMR, and EER

We further used False Match Rate (FMR), False Non-Match Rate (FNMR), and Equal Error Rate (EER) to measure the accuracy of the fingerprint recognition system. FMR, also referred to as False Acceptance Rate (FAR), is the percentage of impostor/unmatched comparisons that are incorrectly accepted. FNMR is the percentage of genuine/matched comparisons that are incorrectly rejected. FMR and FNMR, are computed using the similarity score as,

(19) $$\begin{array}{r} FMR=\frac{N_{AI}}{N_{I}}\\ FNMR=\frac{N_{RG}}{N_{G}} \end{array}$$

#### ROC–Receiver Operating Characteristics

**Figure 10:**
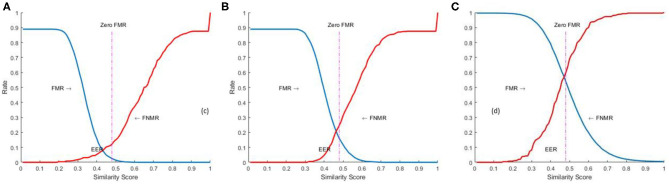
FMR and FNMR (FVC2004, 2012) for a given threshold (t) **(A)** FVC2002 **(B)** FVC2004 **(C)** NIST SD27.

Where “N_AI_” is the number of accepted imposters, “N_I_” is the number of imposter attempts, “NRG” is the number of rejected genuine, and “NG” is the number of genuine attempts. EER is the error rate at the threshold “t” when FMR = FNMR. The system performance is also reported at all operating points (threshold t), by plotting FMR (t) against FNMR (t) as a receiver operating characteristic (ROC) curve as shown in Figure 10.

**Figure 11:**
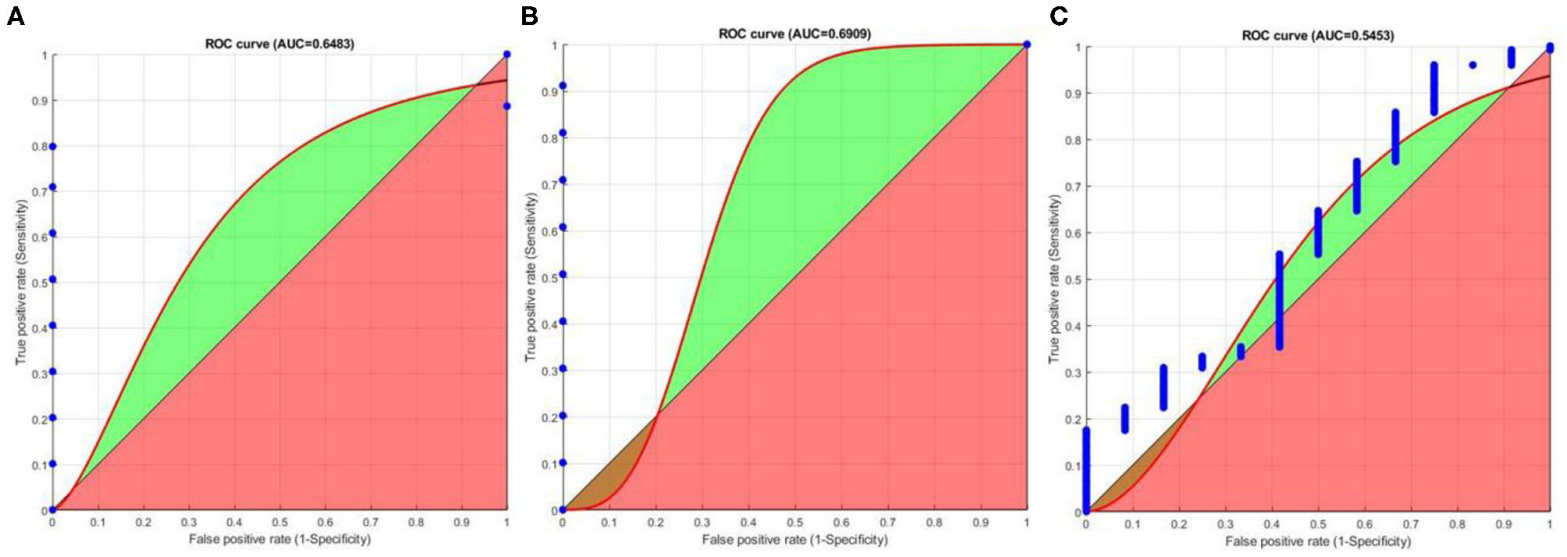
AUROC graphs (Cardillo, 2008) to measure performance FEM for, **(A)** FVC2002 **(B)** FVC2004 **(C)** NIST SD27 datasets.

We plot ROC (Cardillo, 2008) graphs for organizing a binary classifier and to visualize their performance. ROC graphs are commonly used fingerprint recognition systems. Sensitivity is defined as the probability that a test result will be positive when the true fingerprint is present (true positive rate). Specificity is the probability that a test result will be negative when the true fingerprint is not present (true negative rate). Area Under Curve (AUC) ROC also known as “AUROC” is used to check the classification model's performance. Here, ROC is a probability curve and AUC represent the degree or measure of separability. It tells us how much the model is capable of distinguishing between classes. The higher the AUC, the better the model is at predicting “0” s as “0” s and “1” s as “1” s. By analogy, the higher the AUC, the better the model is at distinguishing between the true fingerprint and false fingerprint. We use the function to compute and plot the AUROC curves for Sensitivity, and Specificity (See Figure 11). From Figures 11A,B, it can be observed that FEM can easily distinguish between the true and false fingerprints of FVC2002 and FVC2004 databases. However, for the NIST SD27 database, the AUC is about 0.55 indicating FEM has a discrimination capacity of about 50%. This is because the NIST SD27 is a latent fingerprint database and the quality of fingerprints is very poor.

## Conclusion and Future Scope

We have proposed a fully automated end-to-end latent fingerprint identification system. We initially enhanced the low-quality fingerprints using a DCNN model and further enhancement was achieved using an FFT enhancement algorithm. This enhancement resulted in an improved ridge structure even after skeletonization. Latent enhanced images were extracted using the Automatic Latent Minutiae Extractor (ALME) and the final match results were obtained by Frequency Enhanced Minutiae Matcher (FEMM). ALME was able to extract a good number of minutiae with a smaller number of tolerable false minutiae. In spite of this, our matcher achieved a precision of 87.88 and 76.19% for FVC2004 and NIST SD27 databases, respectively. We obtained a Rank-1 identification accuracy of 100% for FVC2002/2004 datasets, and 84.5% for all latent prints in the NIST SD27 dataset. We compared matching results with good, bad, and ugly laments separately. We compared the performance of latent enhancement, extraction, and matching results with the state-of-the-art methods for FVC2002, FVC2004, and NIST SD27 databases. Our proposed enhancement, extraction, and matching methods performed well compared to the state-of-the-art methods.

The system is not invariant to rotation and scale. To improve the robustness of the system we can develop a scale-rotation invariant based minutiae matcher similar to the method proposed by Deshpande et al. (2020b). A deep minutiae extractor model “MINU-EXTRACTNET” Deshpande and Malemath (in press) can be utilized along with the proposed FFT enhancement module to improve the systems matching accuracy. To reduce the computational cost of matching, we can integrate a hash-based indexing system for faster retrieval. We used the pre-trained model (Tang et al., 2017), trained with a total of 8,000 images including plain (3,200 images) and augmented images from the FVC 2002 dataset. To improve the learning, we can train the models for additional latent databases. To improve the overall performance of the system, we can develop and integrate all the modules of the identification system using a deep network.

## Data Availability Statement

All datasets generated for this study are included in the article/supplementary material.

## Author Contributions

UD and VM were involved in analyzing and implementing the algorithms. SP and SC were involved in organizing the dataset and reporting results. All authors contributed to the article and approved the submitted version.

## Conflict of Interest

The authors declare that the research was conducted in the absence of any commercial or financial relationships that could be construed as a potential conflict of interest.

## Footnotes

^1^*NIST Fingerprint Minutiae Viewer (FpMV)*. Available online at: https://www.nist.gov/services-resources/software/fingerprint-minutiae-viewer-fpmv.
